# Iatrogenic Arteriovenous Fistula: A Rare Complication of Pacemaker Implantation

**DOI:** 10.1016/j.jaccas.2021.11.022

**Published:** 2022-04-06

**Authors:** William Lee, Jason K. Wong, Erkan Ilhan, Satish R. Raj, Vikas P. Kuriachan

**Affiliations:** a Department of Cardiac Sciences, Libin Cardiovascular Institute, University of Calgary, Calgary, Alberta, Canada; b University of New South Wales, Sydney, New South Wales, Australia

**Keywords:** bradycardia, cardiac pacemaker, complication, AVF, arteriovenous fistula

## Abstract

Severe vascular complications associated with pacemaker implantation are rare. Typically, they are overt, and require immediate resolution. We present 2 patients with insidious presentation of arteriovenous fistulae due to pacemaker implantation that were recognized early post-implantation. Both were repaired endovascularly and had good outcomes post-repair. (**Level of Difficulty: Intermediate.**)

## Central Illustration

**Figure:**
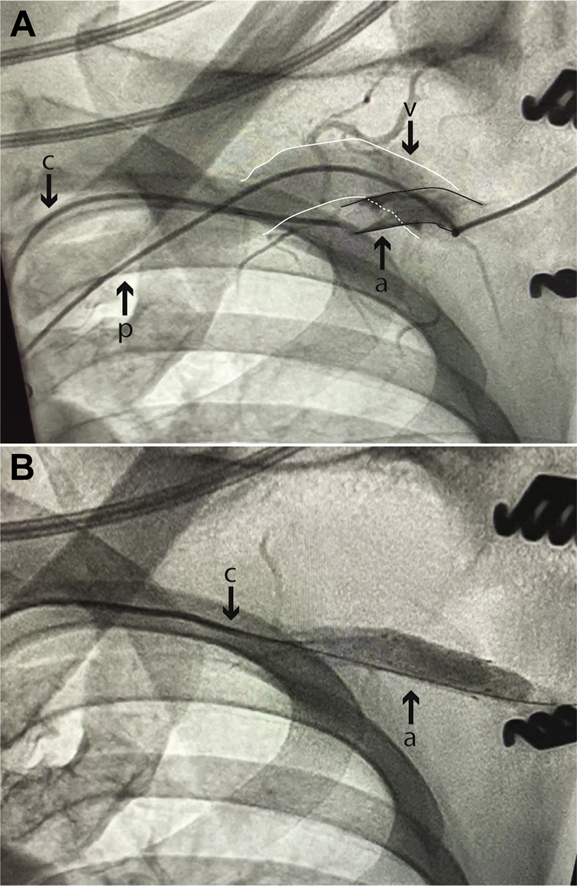

The precise number of vascular injuries associated with cardiac device implantations is unknown and is likely under-reported in the literature. Often these complications are overt and require immediate resolution (eg, arterial bleeding). We present 2 patients presenting with arteriovenous fistula (AVF), as a result of pacemaker implantation. Both were insidious in nature, with variable delays in recognition and treatment. Both patients had good clinical outcomes. We discuss the importance of early recognition of this complication of pacemaker implantation and methods to repair the vascular injury, as well as surgical techniques to avoid such complications.

**Learning Objectives**

• To identify signs and symptoms associated with AV fistulae as a result of cardiac device implantation.
• To examine endovascular repair techniques used to repair iatrogenic AV fistulae.
• To understand surgical techniques to avoid creation of AV fistulae.

## Case 1

A 77-year-old male was referred to our institute by his local cardiologist for an incidental chest bruit, loudest in the left subclavicular region. The patient was otherwise clinically stable.

One year prior, he had undergone a bioprosthetic mitral valve replacement and tricuspid annuloplasty for severe degenerative mitral and tricuspid regurgitation, complicated by atrioventricular conduction block requiring ventricle-ventricle inhibited pacemaker implant.

A computed tomography chest scan with contrast was performed, which showed that the pacing lead had passed through the left axillary artery to the left axillary vein, suspicious for the presence of an AVF.

**Figure 1:**
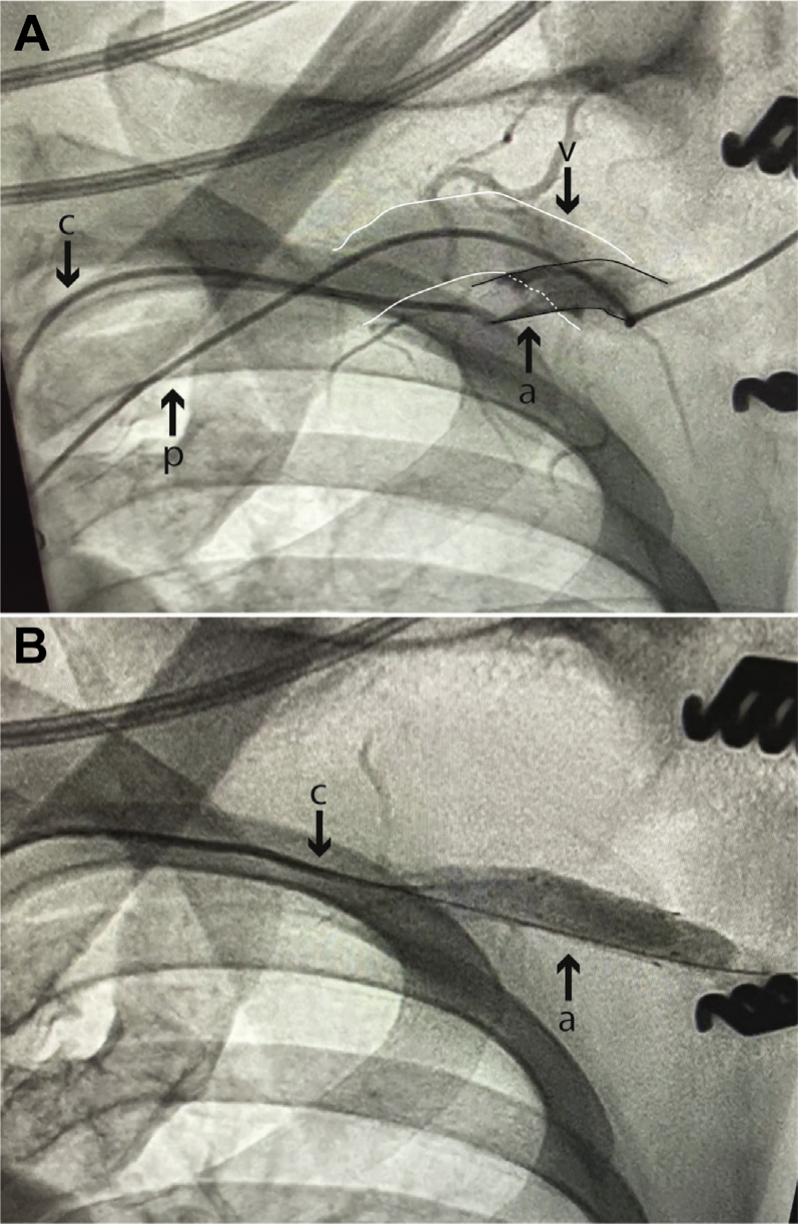
Case 1 **(A)** Injection of contrast from a femoral artery catheter (c) into the left subclavian artery (a) **(outlined in black)** with opacification of the left subclavian vein (v) **(outlined in white)** (which runs along the tract of the pacing lead [p]) via an arteriovenous fistula. **(B)** Post-extraction of the pacing lead and deployment of a covered stent. Injection of contrast in the left subclavian artery shows no further flow seen in the fistula and no opacification of the left subclavian vein.

The patient was taken to the operating room, where angiography confirmed the AVF communication (Figure 1A, Video 1).

The pacemaker lead was removed with simple traction. Given the large caliber of the artery an 8 mm × 2.5 cm covered stent (Viabahn, Gore Medical) was deployed at the level of the AVF. Follow-up axillary angiography (Figure 1B, Video 2) showed no further AVF communication, with immediate resolution of the patient’s bruit. Pacemaker interrogation showed late recovery of atrioventricular conduction with negligible ventricular pacing. A decision was made to not re-implant a new pacemaker.

## Case 2

A 63-year-old male underwent implant of a dual-chamber pacemaker for high-grade atrioventricular conduction block. The procedure was complicated by difficult venous access, requiring venogram guidance and multiple punctures with an introducer needle to successfully cannulate the subclavian vein. Significant bleeding around lead insertion sites was noted during the procedure, and several purse-string sutures were applied in the pocket to achieve hemostasis.

**Figure 2:**
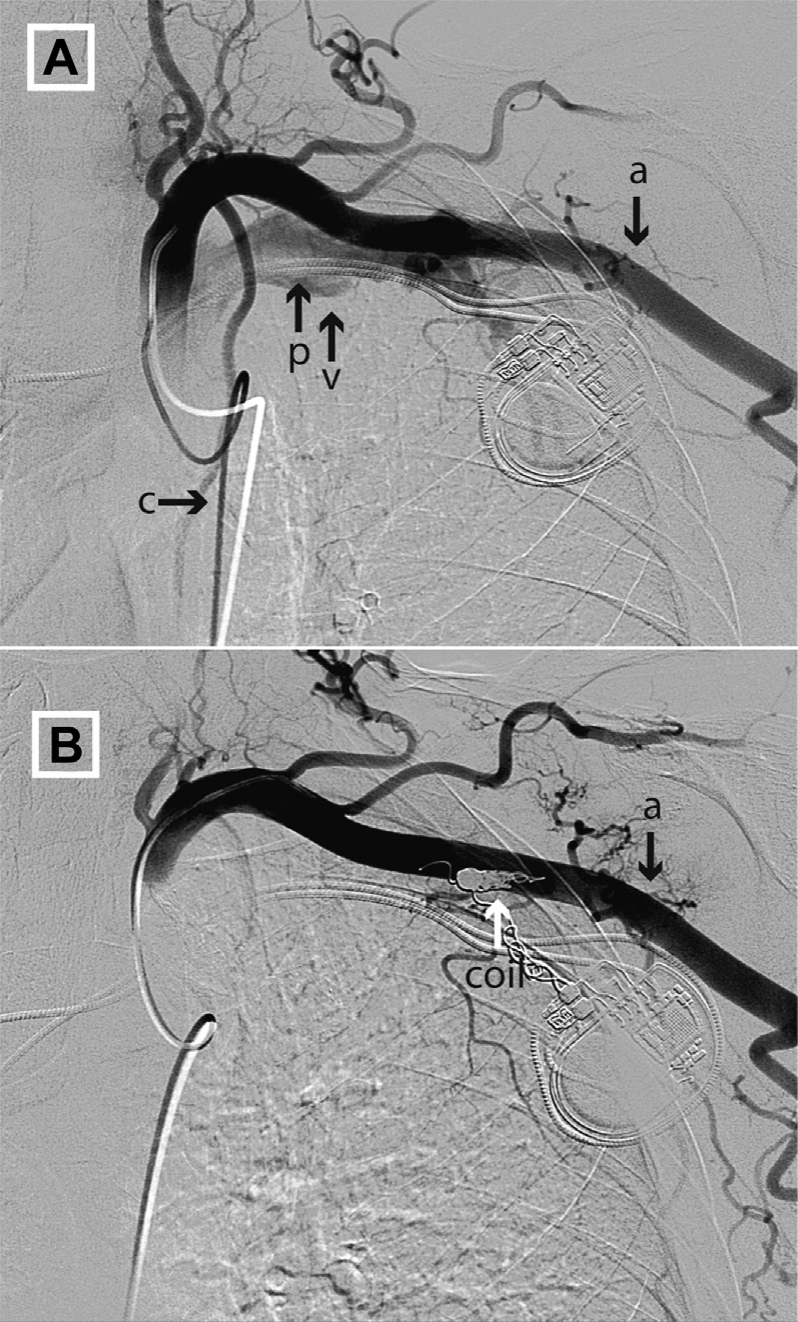
Case 2 **(A)** Injection of contrast with digital subtraction angiography from a femoral artery catheter into the left subclavian artery. The left subclavian vein (which runs along the tract of the pacing lead) fills via an arteriovenous fistula from a small branch of the subclavian artery. **(B)** 2 endovascular coils are deployed in the proximal part of the branch vessel, occluding it and preventing blood flow into the subclavian vein via the fistula. Abbreviations as in Figure 1.

At his 6-week follow-up appointment, a loud chest bruit was noted. A computed tomography chest scan with contrast showed the presence of an AVF related to pacemaker lead insertion. Digital subtraction angiography confirmed an AVF with a small branch of the subclavian artery filling the subclavian vein (Figure 2A, Video 3).

Given the small caliber of this branch vessel and its origin distant from the pacing lead entry site, a microcatheter and microwire were used to successfully deliver 2 Penumbra Ruby detachable coils 4 mm × 15 mm (Penumbra Inc) at the proximal portion of the branch artery. Further angiography showed no further flow through the AVF or opacification of the subclavian vein (Figure 2B, Video 4). The bruit was no longer present at the end of the procedure.

## Discussion

AVF post-pacemaker implantation is extremely rarely reported in the literature.^1^ No large case series exists to reveal the true incidence of this complication. In this limited literature, symptoms such as excessive bleeding during implantation or ipsilateral arm swelling should raise suspicion that an arteriovenous vascular injury has occurred.^2^ Late complications may include chronic venous insufficiency or ischemia of the affected upper limb, or high-output heart failure.^3^ Our patients, in contrast, were asymptomatic suggesting a smaller volume of blood shunting via the AVF.

Both cases were discovered early post-implantation. Therefore, the use of covered stents or coils to occlude the AVF is highly successful with low morbidity, and it is the preferred method over open surgical repair, given its significantly lower risk of morbidity/mortality compared to open vascular repair.^4^ If the pacing lead is in close proximity to the site of embolization or stent deployment (as in Case 1 but not Case 2), we suggest that the pacing lead first be extracted before deployment of the stent or coil, as “jailing” of the pacing lead is a potential risk if endovascular repair is performed with the pacemaker in situ. Jailing the pacing lead may damage the pacing lead itself. Additionally, jailing the lead or late recognition of the fistula would make a future pacing lead extraction technically more difficult, and may require an open surgical approach as the use of powered extraction tools may result in arterial injuries resulting in more catastrophic complications such as limb infarction or hemothorax.

**Figure 3:**
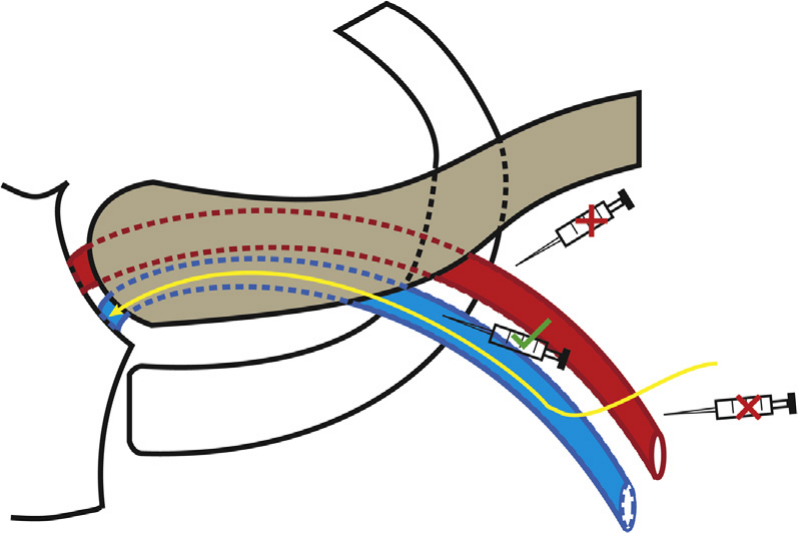
Venipuncture During venipuncture, the artery can be encountered when taking a cranial to caudal approach or a lateral approach as depicted by the **red-cross** syringe icons. The ideal puncture site is near the outer border of the first rib, as depicted by the **green tick** syringe icon. The tract of the pacing lead in our reported case 1 is shown in **yellow**.

AVF injuries are more likely when performing an axillary vein puncture using either a cranial-to-caudal or an extreme lateral approach with the introducer needle (Figure 3). The ideal puncture site should be the region of the outer border of the first rib.^5^ The use of ultrasound guidance, compared to fluoroscopy with venography, may allow the operator a better appreciation of the arteriovenous anatomical relationship and reduce the risk of arterial injuries.^6^ Alternatively, surgical cutdown approaches under direct visualization (eg, cephalic vein) may reduce this risk for AVF.

## Conclusions

Iatrogenic AVF is a rare complication of pacemaker implantation surgery. Endovascular repair of AVF is a possible option for cases that are recognized early.

## Funding Support and Author Disclosures

Dr Wong has been speaker for Boston Scientific, Cook Medical, and Medtronic; and is on advisory boards for Eisai and Astra Zeneca. Dr Raj has received honoraria from the Academy for Continued Healthcare Learning for developing CME slides kits on neurogenic orthostatic hypotension; is a Data and Safety Monitoring Board Chair for a Phase 2 study of an irritable bowel syndrome medication for Arena Pharmaceuticals with compensation; and has received consulting fees from Lundbeck LLC and Theravance Biopharma related to neurogenic orthostatic hypotension. Dr Kuriachan has received honorarium and been on the advisory board for Medtronic, Bayer, Bristol Myers Squibb, and Servier; and has received grants from Abbott, Novartis, Servier, and Medtronic. All other authors have reported that they have no relationships relevant to the contents of this paper to disclose.

## Appendix

Supplemental Video 1 Case 1. Contrast is injected into the left subclavian artery as the catheter is slowly withdrawn. Initially, this opacifies the left subclavian artery. As the catheter is withdrawn, the fistula tract and left subclavian vein are opacified which both run along the course of the pacing lead.

Supplemental Video 2 Case 1. Post-extraction of the pacing lead and deployment of a covered stent. Catheter and guidewire via the right femoral artery are seen in the subclavian artery. Injection of contrast in the left subclavian artery shows no further flow of contrast via the fistula and no opacification of the left subclavian vein.

Supplemental Video 3 Case 2. A catheter is placed in the left subclavian artery via the right femoral artery. Digital subtraction angiography is used to show opacification of the left subclavian artery, branch artery, and vein via a pacing lead fistula.

Supplemental Video 4 Case 2. The pacing leads are not extracted in this case. Endovascular coils are deployed into the involved branch artery. Digital subtraction angiography shows successful endovascular coil occlusion of the branch artery with no further fistula flow into the subclavian vein.
